# Sensitive Spectral and Temporal–Spatial Characteristic Analysis of Leaf SPAD in Maize Under Variety and Nitrogen Coupling Effects

**DOI:** 10.1002/fsn3.71907

**Published:** 2026-05-21

**Authors:** Fu Zhang, Baoping Yan, Le Yang, Fangyuan Zhang, Yakun Zhang, Yafei Wang, Shaukat Ali, Sanling Fu

**Affiliations:** ^1^ College of Agricultural Equipment Engineering Henan University of Science and Technology Luoyang China; ^2^ College of Mechatronics Engineering Henan University of Science and Technology Luoyang China; ^3^ College of Agricultural Engineering Jiangsu University Zhenjiang China; ^4^ Wah Engineering College University of Wah Wah Cantt Pakistan; ^5^ College of Physics Engineering Henan University of Science and Technology Luoyang China

**Keywords:** dynamic growth effects, maize, SPAD, temporal–spatial characteristics, vegetation indices

## Abstract

Accurate estimation of leaf SPAD is crucial for maize growth and yield formation. Many methods for monitoring SPAD currently lack the analysis of sensitive leaf position in different stages of maize. In this paper, the spectra and temporal–spatial characteristics of maize leaf SPAD were analyzed to describe the sensitive stage and leaf position. After exploring the dynamic growth effects of SPAD in maize leaves, the sensitive stage of SPAD was determine. Several preprocessing methods and spectral vegetation indices were used to analyze the spectral reflectance of typical leaf positions in sensitive stages. The function regression methods based on single vegetation index and the random forest regression (RFR) based on multi‐vegetation indices were employed. The results showed that the twelve‐leaf (V12) and the silking (R1) were the sensitive stages. The strongest RVI at the V12 stage and NDRE for the ear leaves at the R1 stage were observed under SG‐SNV method. The best prediction data (*R* ^2^ = 0.7) was showed at the V12 stage under MSC‐RF. The prediction effect of the ear leaves after MSC pretreatment was slightly better (*R* ^2^ = 0.69). In addition, SPAD value can indirectly reflect the chlorophyll content, nitrogen content and yield status of maize leaves, and its accurate monitoring provides effective guidance for maize leaf nutrition information and yield prediction.

## Graphical abstract

The data processing flow was shown in graphical abstract. The first phase was construction of dataset, including acquisition and inordinate value elimination of spectral data and SPAD of maize leaves. The second phase was the dynamic growth effect analysis of SPAD value of maize leaves at different growth stages and leaf positions. The third phase was characteristic analysis, including the spectral vegetation characteristics, temporal–spatial characteristics of leaf SPAD. The fourth part was construction of SPAD prediction model.

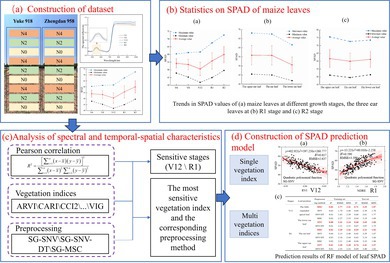

## 1 Introduction

Maize is considered an important crop providing food, feed, energy, and other raw materials for global industries (Chen et al. 2024; Liu et al. 2024). Leaves serve as the primary organs responsible for photosynthesis and nitrogen storage in maize plants, their life span is the duration from full expansion of leaves to senescence and abscission (Nürnberg et al. 2018). The leaf chlorophyll content (LCC) is a significant indicator of plant photosynthetic capacity and growth conditions, and many studies have demonstrated that LCC is directly related to the final yield of maize (Tsele et al. 2023; Pan et al. 2025; Wang et al. 2021). Traditional approaches for the quantification of chlorophyll content mainly include acetone ethanol extraction, spectrophotometry and high‐performance liquid chromatography. Such destructive methods based on laboratory procedures are time consuming, expensive, and not suitable for high‐throughput phenotyping (Gholizadeh et al. 2017). The soil and plant analyzer development (SPAD) manufactured by Konica Minolta in Tokyo, Japan, is used to measure the relative chlorophyll content in leaves (Yuan et al. 2016). Compared with traditional methods, the implementation of SPAD tool has numerous advantages, including enhanced efficiency, nondestructive operation, and freedom from time and environmental constraints (Chen et al. 2023; Wu et al. 2024). Therefore, SPAD can be selected as a representative measurement for LCC, aiding in assessing plant nitrogen nutrition status and scientifically guiding nitrogen fertilizer application rates, thereby ensuring maize yield and quality.

In‐situ hyperspectral technology has significant advantages, including strong spectral continuity, high spectral resolution, and rich spectral information, which renders it highly applicable in the field of crop information sensing (Fu et al. 2021). Hyperspectral technology collects reflected spectral energy from crop leaf surfaces, which can provide reflectance data in the visible (VIS), red edge (RE), and near‐infrared (NIR) regions. LCC primarily resides within the visible light spectrum (Huang et al. 2023; Li, Wijewaedane, et al. 2023; Xiao et al. 2022; Zhao et al. 2023). Consequently, hyperspectral technology is suitable for the quantitative monitoring of chlorophyll levels within crop leaves.

In the estimation of crop chlorophyll levels, the methods employed have evolved significantly from an initial reliance on multiple regression techniques to the predominant use of empirical/semi‐empirical spectral index methods. Meanwhile, some researchers have used physical modeling methods for the inversion. However, the complexity of the inversion algorithms and the numerous uncertainties involved in physical modeling made it difficult to obtain many input parameters, resulting in limitations on inversion accuracy (Liu et al. 2021). In contrast, the inversion methods based on spectral indices derived from linear or nonlinear combinations of two or more spectral bands are more sensitive to spectral information than single‐band approaches. Hyperspectral vegetation indices have been shown to enhance spectral sensitivity to chlorophyll through narrow‐band combinations, thereby reducing the issue of band overfitting caused by excessive band usage (Chen et al. 2010; Rajeev et al. 2012). A significant number of scholars have proposed sensitive hyperspectral vegetation indices based on maize chlorophyll for non‐destructive estimation of leaf or canopy chlorophyll indicators. Vegetation indices were constructed using maize canopy spectral data acquired by drones, and SPAD value prediction models were developed for maize leaves based on these indices. The results showed that indices such as WDRVI, ARVI, NDRE, MCARI, and CIrededge were sensitive to chlorophyll levels at certain growth stages or nitrogen application rates (Parida et al. 2024; Ma et al. 2024; Su et al. 2023).

In addition to utilizing a solitary hyperspectral vegetation index as an indicator of chlorophyll level changes, machine learning methods have the capacity to concurrently contemplate multiple hyperspectral features that are sensitive to change, and serve to enhance the accuracy of chlorophyll level estimation. Common modeling methods include partial least squares regression (PLSR), random forest regression (RFR) and support vector regression (SVR) (Yao et al. 2025). Lopez‐Calderon et al. (2020) found that the existing vegetation indices as the input variable of the RF algorithm had a better performance in estimating the nitrogen content of maize. Zhang et al. (2022) used three spectral indices extracted from hyperspectral imagery—the Quantitative Vegetation Index (QVI), the Normalized Difference Vegetation Index (NDVI), and the Modified Chlorophyll Absorption Ratio Index (MACARI)—combined with PLSR model to predict leaf chlorophyll content.

The majority of extant studies analyze maize canopy leaf information. In contradistinction to other crops, maize plants exhibit tall growth habits with numerous leaves that spread widely. The fact that nitrogen is easily transported results in a distinct vertical gradient of leaf nitrogen nutrition indicators throughout the plant, which has varying effects on SPAD values in leaves at different positions on the plant (Li, Sainan, et al. 2022). A plethora of studies have examined the effects of different leaf positions on nutrient levels, revealing that the spatial distribution of SPAD in maize leaves exhibit typical bell‐shaped vertical heterogeneity under varying nitrogen levels (Li, Chang, et al. 2022). As the core organ of maize growth and yield formation, the fully expanded apical leaves have the functions of energy manufacturing, material transportation and physiological regulation. During the vegetative growth stage, the fully expanded apical leaves maximize light exposure, enhancing overall photosynthesis, promoting stem elongation, and storing nutrients for subsequent tassel and ear differentiation. During the reproductive growth stage, the three ear leaves serve as the primary fully expanded apical leaf, responsible for supplying nutrients to the kernels and maintaining root vitality. Consequently, the nutritional monitoring of the fully expanded apical leaves during each growth stage requires further investigation, enhancing the accuracy and validity of leaf SPAD spectral inversion model.

Based on field corn experiment under the coupled effect of variety and nitrogen application rate, the study was conducted to analyze the dynamic growth effects of SPAD and spectral data in maize leaves at different growth stages and leaf positions, revealing the overall SPAD trends throughout the maize growth cycle and the spectral characteristics of leaves at different positions during various growth stages, exploring the intrinsic relationship between SPAD and spectral reflectance. The SPAD prediction model for maize leaves was constructed based on hyperspectral data, enabling the selection of optimal SPAD sensitive temporal–spectral features. This approach holds significant implications for precise nutrient diagnosis and management during critical growth stages of crops.

## 2 Materials and Methods

### 2.1 Field Experimental Materials and Design

**FIGURE 1:**
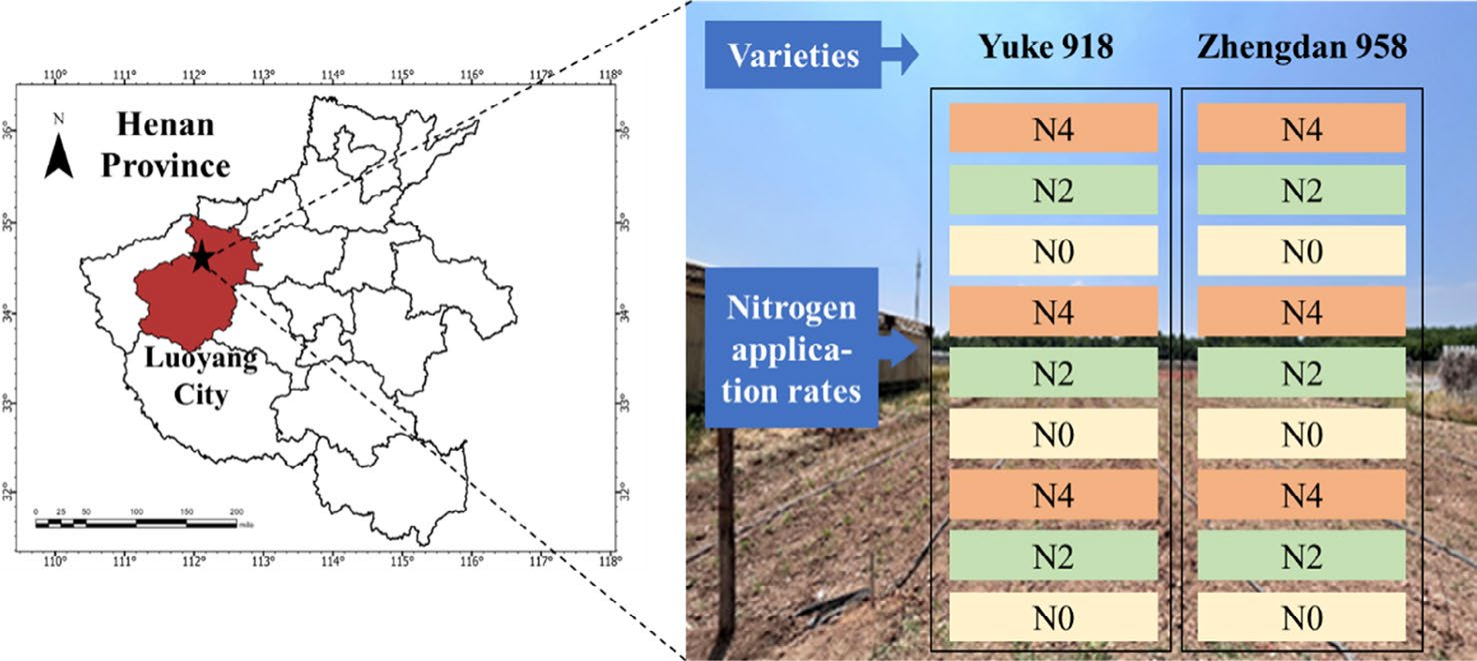
Location and arrangement of the experimental plots.

The experimental design was illustrated in Figure 1. The varieties Zhengdan 958 (Institute of Food Crops, Henan Academy of Agricultural Sciences, P. R. China) and Yuke 918 (Henan University of Science and Technology, P. R. China), renowned for their excellent quality, adaptability and stress tolerance, were selected for this study. The experiment involved three distinct nitrogen application rates: 0, 200, and 400 kg/hm^2^, respectively denoted as N0, N2, and N4. Each experimental plot measured 3.5 m × 13.5 m, with phosphorus fertilizer (P_2_O_5_) and potassium fertilizer (K_2_SO_4_) applied at 150 kg/hm^2^, both administered in a single application at the time of sowing. The irrigation system utilized was of the drip irrigation type, with equal row spacing and a planting density of 75,000 plants per hectare. The sowing process was initiated on 1 May 2024, with the harvesting of the crop taking place on 31 August 2024. All other management practices adhered to field management guidelines for maize.

### 2.2 Description of the Experimental Dataset

#### 2.2.1 Acquisition of Leaf Spectral Data and SPAD

The ASD Field Spec Hand Held 2 spectroradiometer (Analytical Spectral Devices Inc., Boulder, CO, USA) was utilized to measure the spectral reflectance of the leaves, the effective wavelength range of which was 325–1075 nm, with a sampling interval of 1 nm, spectral resolution of 3 nm, and a field of view of 25°. In conditions of clear weather and minimal wind, hyperspectral data was collected from fully expanded apical leaves during the vegetative growth stages (six‐leaf stage, V6; eight‐leaf stage, V8; twelve‐leaf stage, V12) and the three ear leaves (the upper ear leaf, the ear leaf, the lower ear leaf) during the silking (R1) and grain‐filling (R2) stages. The phenological stages and leaf positions involved in the maize samples are shown in Table 1. Before spectral acquisition, the standard whiteboard correction was performed, and the veins were avoided during the measurement. Each leaf was repeatedly measured three times and marked. The average spectral reflectance of the three times was taken as the final spectral reflectance of the leaf.

**TABLE 1:** Phenological stages and leaf positions involved in the maize samples.

The phenological stages	Introduction	Typical leaf position
The six‐leaf stage (V6) The eight‐leaf stage (V8) The twelve‐leaf stage (V12)	The sixth/eighth/twelfth true leaf on the maize plant is fully expanded, and its leaf pillow (leaf ear) is clearly visible.	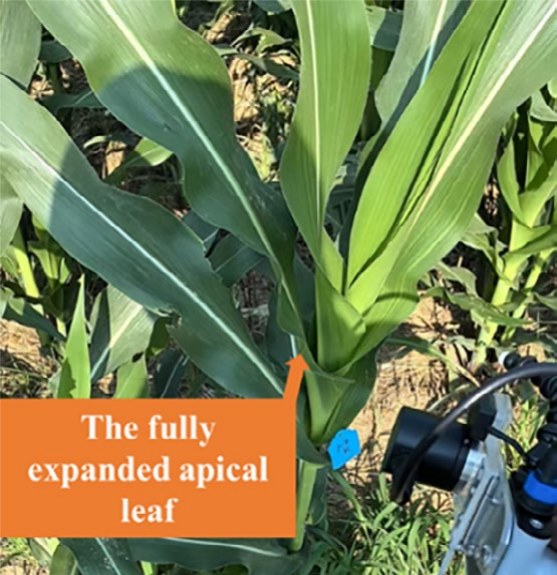
The silking stage (R1)	The filaments on the ear of maize protrude from the bracts.	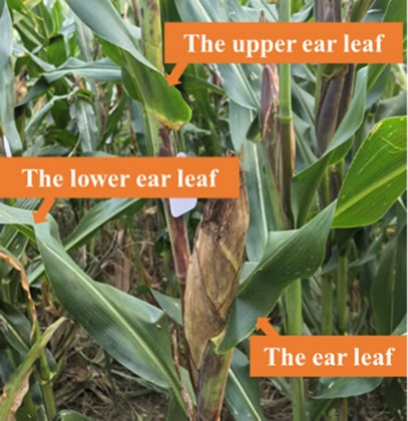
The grain‐filling stage (R2)	After the pollination of maize, the stage of nutrient transport to the grain, the grain becomes full and accumulates dry matter.	

SPAD measurements of maize leaves were taken using SPAD‐502Plus (Konica Minolta, Tokyo, Japan) chlorophyll meter, synchronized with hyperspectral data acquisition to ensure measurement locations aligned with hyperspectral data collection points. Each leaf was the subject of three repeated measurements, with the mean value recorded as the leaf SPAD.

#### 2.2.2 Construction of the Experimental Dataset

In the course of data acquisition, the anomalous data resulting from instrumentation or manual operation had the potential to compromise the model performance in a predictive capacity. Consequently, the K‐means clustering and Euclidean distance were employed to eliminate abnormal SPAD value. Meanwhile, PLSR coupled with the Monte Carlo method was used to remove the anomalous samples from the spectral datasets.

As a classical unsupervised learning algorithm, K‐means clustering assumed that the distance between the data point and the clustering center obeyed the normal distribution, and defined the data point whose distance was greater than the sum of the average distance and twice the standard deviation as the outlier (Pan et al. 2024). Monte Carlo method was a calculation method based on random sampling. PLSR, as a regression method suitable for high‐dimensional data, could effectively deal with the multicollinearity problem of spectral data. Based on multiple random sampling, the PLSR model was used to fit the data to evaluate the stability of the data points, and the abnormal spectral samples were identified by residual analysis (Fan et al. 2025). The specific operation steps were as follows:

1. The maximum of components was set to 20, and the best PLSR component number was selected by 10‐fold cross‐validation.
2. The number of iterations N of Monte Carlo method was set to 1000, and the threshold of outliers was set to three times the standard deviation. The initial PLSR model was fitted with the optimal number of components, and the initial residual was calculated.
3. N random samplings were performed, the PLSR model was fitted. The residuals were calculated, and the samples with residuals exceeding the threshold were marked as outliers.

In order to ensure concordance between spectral data and SPAD values, spectral data associated with anomalous SPAD values and SPAD values linked to anomalous spectral data were simultaneously excluded. The final data for different periods and leaf positions were presented in Table 2. The minimum variation range for SPAD values in maize leaves was 41.9–51.4, while the maximum variation range was 60.4–75.5. The coefficient of variation, being a dimensionless metric, indicated that a smaller coefficient signifies a more concentrated and relatively stable distribution of data points. The coefficient of variation for each dataset remained below 10%, which can meet characteristics requirements.

**TABLE 2:** Statistical characteristics of SPAD values.

Stages	Leaf position	Number of samples	Maximum value	Minimum value	Average value	Standard deviation	Coefficient of variation /%
V6	The fully expanded apical leaf	225	60.4	47.9	54.2	3.0	5.5
V8	The fully expanded apical leaf	219	61.2	45.3	53.5	3.4	6.3
V12	The fully expanded apical leaf	228	61.6	41.9	50.6	4.3	8.4
R1	The upper ear leaf	227	68.1	45.5	55.9	5.3	9.5
	The ear leaf	228	67.5	45.0	55.8	5.3	9.5
	The lower ear leaf	228	61.6	41.9	50.6	4.3	8.4
R2	The upper ear leaf	224	75.5	51.1	61.7	5.8	9.5
	The ear leaf	228	72.6	49.6	60.0	5.2	8.7
	The lower ear leaf	229	73.5	51.4	61.2	5.0	8.1

SPAD value could indirectly reflect the LCC, and then characterize the nitrogen nutrition level of maize plants. The internal mechanism is that the chlorophyll molecular structure contains a large amount of nitrogen, and the leaf nitrogen content is significantly positively correlated with chlorophyll synthesis. When the nitrogen availability in soil or medium is high and the supply is sufficient, maize can synthesize more chlorophyll, and the SPAD value increases accordingly. On the contrary, nitrogen deficiency will limit chlorophyll synthesis, resulting in a decrease in SPAD value. The typical symptom of leaf nitrogen deficiency is that the leaf color gradually becomes lighter from normal dark green, first showing light green, and then developing into yellow. In severe cases, the leaves are dry and dry, and lose photosynthetic function. However, the absolute SPAD value can only reflect the LCC of a single sample, its value is greatly affected by non‐nitrogen factors such as maize variety, environment, growth stage and leaf position deviation, and cannot simply correspond to the nitrogen supply level. Therefore, the relative SPAD value (RS, the ratio of SPAD value in the test area to SPAD value in the high nitrogen control area) is used as a reference to guide the nitrogen supply management, eliminate the differences between varieties and environment. RS < 0.95 indicates that the sample area is short of nitrogen and needs topdressing. RS ≥ 0.95 indicates that the sample area had sufficient nitrogen and no topdressing (Shivashankar et al. 2025; Martínez et al. 2025).

### 2.3 Description of Data Analysis

#### 2.3.1 Spectral Data Preprocessing for Maize Leaves

During the acquisition of spectral data, extraneous information such as noise, background interference and stray light could influence the results of subsequent spectral analysis. Therefore, data preprocessing was required to mitigate random noise and enhance the signal‐to‐noise ratio. The Savitzky–Golay (SG) convolution smoothing method was employed to enhance the smoothness of spectral curves (Massaoudi et al. 2020). The standard normal variate transformation (SNV) effectively eliminated spectral errors caused by the factors such as the baseline drift and the variations in the optical path length through the standardization processing, which improved data comparability and enhanced model performance. Detrend (DT) was a method of preprocessing spectral data that eliminated baseline drift caused by scattering effects or instrument drift, which was used to effectively amplify the trend of peaks and troughs in the raw spectral curve, thereby preserving valuable spectral information and improving the signal‐to‐noise ratio of the spectral data. Multiplicative Scatter Correction (MSC) primarily addressed scattering effects in spectral data that arose from particle size and surface roughness. By correcting the multiplicative and additive effects in spectral data, the spectral information was made to concentrate more on the intrinsic chemical composition of the material. In summary, three methods (SNV, SNV‐DT, and MSC) were employed in this study for preprocessing raw spectral reflectance database on SG convolution smoothing.

#### 2.3.2 Spectral Vegetation Indices and Correlation Analysis

Vegetation indices, as dimensionless measures describing the growth state of vegetation, were typically calculated through linear or nonlinear combinations of spectral reflectance from two or more bands, reflecting information such as chlorophyll content, photosynthetic efficiency, and growth status within vegetation (Zhao et al. 2023). This approach minimized the influence of background and other factors on spectral characteristics, thereby emphasizing vegetation‐specific information. Twenty‐one vegetation indices sensitive to changes in LCC were selected (Li et al. 2014; Yang et al. 2023; Liao et al. 2023), as shown in Table 3, the vegetation indices were calculated using relevant formulae, based on preprocessed leaf spectral reflectance data.

**TABLE 3:** Vegetation indices formula.

Vegetation indices	Calculation formula
ARVI	(*R*_800_−(2 × *R*_670_−*R*_450_))/(*R*_800_ + *R*_670_ + 0.5)
CARI	(*R*_670_−*R*_450_)−(*R*_670_−*R*_800_)
CCI2	(*R*_800_−*R*_670_)/(*R*_800_ + *R*_670_ + 0.1)
CIgreen	(*R*_800_/*R*_550_)−1
CIrededge	(*R*_800_/*R*_720_)−1
CNDVI	((*R*_800_−*R*_670_)−(*R*_800_−*R*_450_))/((*R*_800_−*R*_670_) + (*R*_800_−*R*_450_))
CVI	*R*_800_^2^/*R*_670_
CVI2	*R*_800_/(*R*_720_ + 1)
DVI	*R*_800_−*R*_670_
EVI	2.5 × (*R*_800_−*R*_670_)/(*R*_800_ + 6 × *R*_670_−7.5 × *R*_450_ + 1)
GNDVI	(*R*_800_−*R*_550_)/(*R*_800_ + *R*_550_)
MCARI	((*R*_670_−*R*_450_)−(*R*_670_−*R*_800_))/*R*_670_
NDRE	(*R*_800_−*R*_720_)/(*R*_800_ + *R*_720_)
NDVI	(*R*_800_−*R*_670_)/(*R*_800_ + *R*_670_)
NLI	*R*_800_/(*R*_670_ + *R*_550_)
PVI	(*R*_800_−*R*_670_)−0.5 × (*R*_800_ + *R*_670_)
RVI	*R*_800_/*R*_670_
SAVI	2.5 × (*R*_800_−*R*_670_)/(*R*_800_ + *R*_670_ + 0.5)
TGI	*R*_550_−0.39 × *R*_670_−0.61 × *R*_450_
VARI	(*R*_550_−*R*_670_)/(*R*_550_ + *R*_670_−*R*_450_)
VIG	(*R*_800_−(2 × *R*_670_ + *R*_450_))/(*R*_800_ + (2 × *R*_670_ + *R*_450_))

*Note:* *Rs* denotes the reflectivity of leaves at wavelength *s*.

Pearson correlation analysis was then performed on vegetation indices discussed and SPAD values using MATLAB software. The strength of correlation between two variables was expressed by the correlation coefficient *r*. It was generally accepted that no correlation exists when |*r*| ≤ 0.3; a weak correlation when 0.3 < |*r*| ≤ 0.5; a significant correlation when 0.5 < |*r*| ≤ 0.8; and a highly significant correlation when |*r*| > 0.8. The calculation of Pearson's correlation coefficient was shown in Formula (1).

(1) $$r_{x,y}=\frac{\sum_{i=1}^{n}\left(x_{i}-\bar{x}\right)\left(y_{i}-\bar{y}\right)}{\sqrt{\sum_{i=1}^{n}{\left(x_{i}-\bar{x}\right)}^{2}}\sqrt{\sum_{i=1}^{n}{\left(y_{i}-\bar{y}\right)}^{2}}}$$

*r*_*x*, *y*_ denoted the correlation coefficients between the variables x and y; *n* denoted the sample size; $\bar{x}$ and $\bar{y}$ denoted the sample means of x and y respectively.

### 2.4 Modeling Methods and Statistical Analysis

#### 2.4.1 Modeling Methods

RFR was a non‐linear regression method based on ensemble learning, capable of effectively handling high‐dimensional data and nonlinear relationships. The subsamples were generated from the original data using the Bootstrap sampling, then employed as training sets to construct decision trees. At each node split within a tree, a subset of features was randomly selected for optimal partitioning, enhancing model diversity and mitigating overfitting (Breiman 2001; Singhal et al. 2024). RFR algorithm contained two important parameters, which represented the number of decision trees and the number of decision tree variables. The search range was 5 to 1000 and 1 to 2/3 feature numbers, respectively. The regression prediction value $\hat{y}$ for RFR could be expressed as Formula (2), where *x*_*i*_ and *y*_*i*_ denoted the input feature, and the target value, *T* signified the number of decision trees, and *f*_*t*_ (*x*) denotes the prediction value of the t‐th decision tree.

(2) $$\hat{y}=\frac{1}{T}\sum_{t=1}^{T}f_{t}\left(x\right)$$

### 2.5 Statistical Analysis

The coefficient of determination (*R* ^2^), root mean squared error (RMSE), and residual prediction bias (RPD) were employed to comprehensively evaluate model performance. The closer *R* ^2^ approached 1, the higher the predictive accuracy of model, the smaller RMSE indicated greater model stability. RPD characterized the predictive capability of model (Table 4). In Formulas ((3), (4), (5)), *x*, $\bar{x}$, *y*, $\bar{y}$, *n*, and SD denoted the predicted value, the mean of the predicted values, the actual value, the mean of the actual values, the sample size and the sample standard deviation.

**TABLE 4:** Relationship between RPD and the predictive ability of the model.

Value	Predictive capability
RPD < 1.0	Extremely poor
1.0 < RPD < 1.4	Poor
1.4 < RPD < 1.8	Acceptable
1.8 < RPD < 2.0	Good
2.0 < RPD < 2.5	Very good
RPD > 2.5	Excellent

(3) $$R^{2}=\frac{{\left[\sum_{i=1}^{n}\left(x-\bar{x}\right)\left(y-\bar{y}\right)\right]}^{2}}{\sum_{i=1}^{n}{\left(x-\bar{x}\right)}^{2}\sum_{i=1}^{n}{\left(y-\bar{y}\right)}^{2}}$$

(4) $$\text{RMSE}=\sqrt{\frac{\sum_{i=1}^{n}{\left(x-y\right)}^{2}}{n}}$$

(5) $$\mathrm{RPD}=\frac{\mathrm{SD}}{\text{RMSE}}$$

## 3 Results and Analysis

### 3.1 Statistics on SPAD of Maize Leaves

**FIGURE 2:**
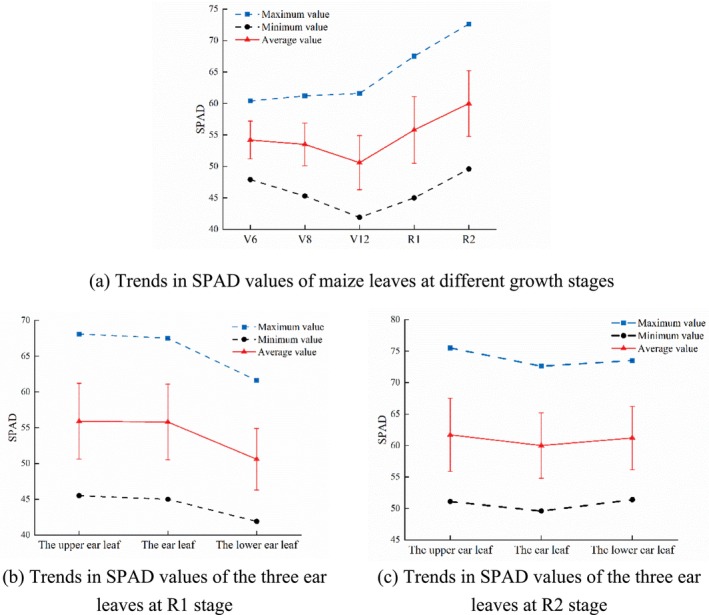
Trends in SPAD values of (a) maize leaves at different growth stages, the three ear leaves at (b) R1 stage and (c) R2 stage.

As shown in Figure 2 (a), SPAD values of maize leaf exhibited a general trend of first decreasing and then increasing from the V6 to R2 stages, reaching a maximum of 72.6 at R2 stage. The SPAD value of the fully expanded apical leaf at V12 stage was found to be the lowest among the five growth stages, at 41.9. A gradual decrease in SPAD value was observed in fully expanded apical leaves as the vegetative growth period progressed. Plants exhibited accelerated growth from the V6 to V12 stages, necessitating an increase in chlorophyll synthesis to facilitate photosynthesis and provide greater nutrients for vertical growth. The alteration in resource distribution resulted in a decline in LCC at V12 stage, SPAD value decreased to the minimum accordingly. Concurrently, the coefficient of variation increased from 5.5% to 8.4%, indicating heightened influence of individual variation and environmental factors on leaf SPAD values.

The statistical data of SPAD values from the three ear leaves at R1 stage was illustrated in Figure 2 (b), which revealed a hierarchical order, with the upper ear leaf exhibiting higher values compared to the ear leaf and the lower ear leaf. The upper leaves were situated near the plant's apex, thus receiving greater light exposure, resulting in enhanced photosynthetic activity, higher chlorophyll content, and elevated SPAD values. Concurrently, these leaves exhibited pronounced individual variation and a higher coefficient of variation due to uneven distribution of light and nutrients. The ear leaves, owing to their proximity to the maize cob, undertook a greater role in nutrient transport, exhibiting markedly enhanced metabolic capacity with SPAD values comparable to those of the upper ear leaves. The lower ear leaves, however, suffered reduced photosynthetic efficiency due to canopy shading, resulting in relatively smaller individual variations and significantly lower SPAD values than both the upper ear leaves and ear leaves.

As shown in Figure 2 (c), the upper ear leaves at R2 stage exhibited high SPAD values due to maximized photosynthetic efficiency under ample canopy light. In comparison with the R1 stage, the SPAD values of the ear leaves increased during the R2 stage, indicating that the functional capacity of these leaves was enhanced to meet the high demand for photosynthetic products. The SPAD values of the lower ear leaves were found to be analogous to those of the upper ear leaves, indicating that the functional capacity of the lower leaves exhibited a recovery during the process of grain filling, thereby providing a synergistic effect that was conducive to the development of the grains. The physiological state of plants stabilized at the R2 stage, in contrast to the R1 stage, with the coefficient of variation for SPAD values in the three ear leaves slightly decreasing.

### 3.2 Correlation Analysis of Spectral Data and SPAD

**FIGURE 3:**
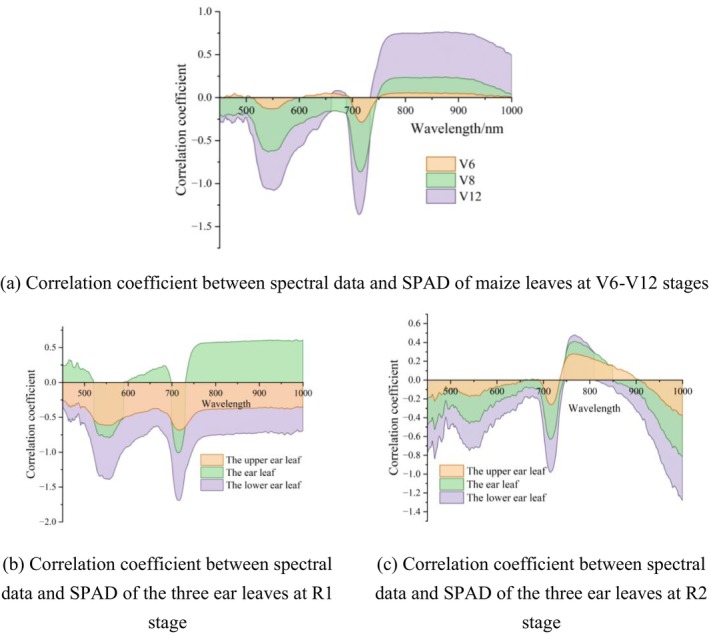
Correlation coefficient between spectral data and SPAD of (a) maize leaves at the V6‐V12 stages, the three ear leaves at (b) R1 stage and (c) R2 stage.

The correlation coefficients between the original spectral reflectance and SPAD values of the fully expanded apical leaf at the V6‐V12 stages increased significantly are shown in Figure 3 (a). The negative correlation was exhibited within the 450–700 nm wavelength range. Within the 700–720 nm red edge region, plant spectra underwent significant transitions, the strong negative correlations were exhibited between spectral reflectance and SPAD values, and reached the zenith at 712 nm (−0.59, V8) and 710 nm (−0.51, V12). This finding suggested that the accumulation of chlorophyll within leaves exerted a growing influence on red edge reflectance as growth progressed. Beyond the 720 nm, the negative correlation gradually weakened and transitioned to the positive correlation. As cellular internal structures underwent change, the correlation stabilized and became positive between 780 and 1000 nm, with the highest correlation coefficient was observed at the V12 stage.

The correlation coefficients between the spectral reflectance and SPAD values of the three ear leaves at R1 stage were demonstrated in Figure 3 (b). Within the 400–1000 nm, both the spectral reflectance of the upper and lower ear leaves exhibited negative correlations with SPAD values. Within the visible light spectrum, the correlations were found to be relatively weak. At the red edge position, the correlation coefficient exhibited a linear increase, shifting from a weak negative correlation to a significant positive correlation. The correlation coefficients in the near‐infrared region demonstrated notable consistency with the red edge position, which exhibited minimal fluctuations and relative stability.

The correlation coefficients between the spectral reflectance of the three ear leaves and the SPAD value are illustrated in Figure 3 (c), which was relatively weak at R2 stage. The negative correlation was observed in the green light region (492–577 nm) and the red light region (622–760 nm), with the stronger negative correlation in the red edge region at 720 nm, which could be attributed to the strong absorption effect of chlorophyll on the red light. The maximum negative correlation coefficient of 0.37 was observed at 711 nm for the ear leaves. The correlation coefficient exhibited the brief positive shift before undergoing a decline to negative values within the 780–1000 nm near‐infrared region. The correlation curve for the upper ear leaves exhibited a significantly higher correlation than that observed for the ear and lower ear leaves, reaching a maximum positive correlation coefficient of 0.28 at 767 nm.

The preceding analysis indicated that the spectral reflectance and SPAD values of fully expanded apical leaves at V12 stage and the three ear leaves at R1 stage exhibited strong correlations, suitable for quantitative chlorophyll monitoring and crop health assessment in precision agriculture.

### 3.3 Correlation Analysis of Spectral Vegetation Indices and SPAD

**FIGURE 4:**
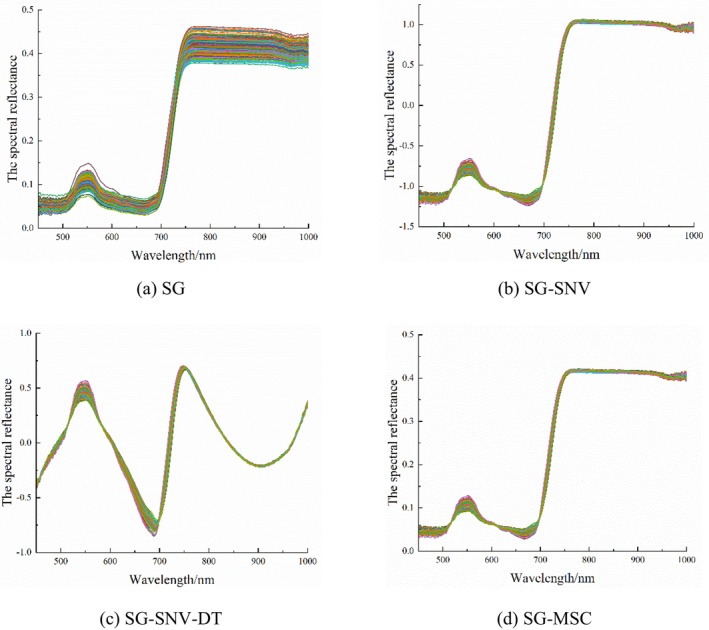
Spectral reflectance curves of different pretreatments.

When acquiring spectral data from maize leaves, interference from various factors were to be expected, including stray light, baseline drift, and instrument dark current. Consequently, spectral preprocessing methods were required to enhance the signal‐to‐noise ratio. Four methods were used to preprocess the raw spectral reflectance of leaves, as were shown in Figure 4.

**FIGURE 5:**
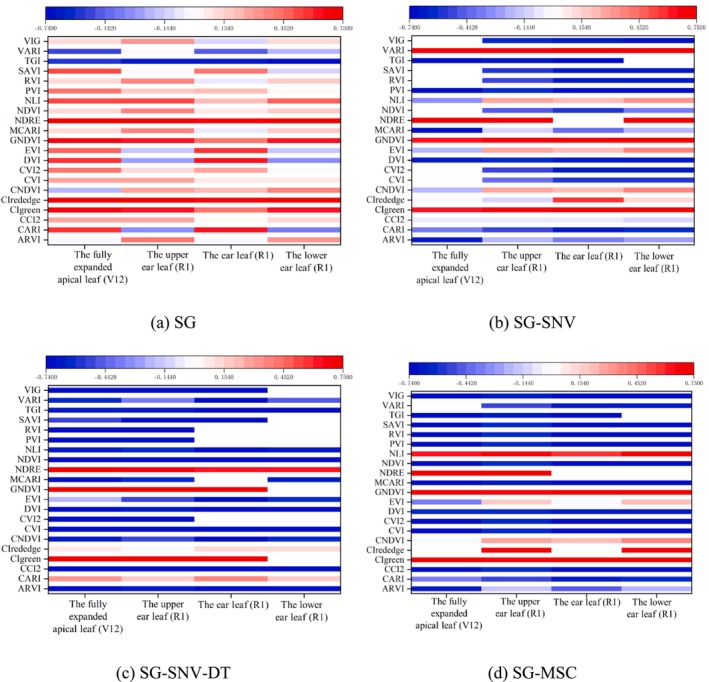
Correlation between spectral vegetation indices and SPAD of different pretreatments.

Spectral reflectance data from the fully expanded apical leaf at the V12 stage and the three ear leaves at the R1 stage were preprocessed to calculate vegetation indices, which were then correlated with SPAD values, as shown in Figure 5. The correlation between vegetation indices preprocessed by different methods and SPAD values exhibited marked differences.

At the V12 stage, the absolute values of the correlation coefficients for the CVI, CVI2, and RVI indices of fully expanded apical leaves with SG‐SNV pretreatment reached a maximum of 0.78. The correlation coefficients for the CIrededge and NDRE indices were maximized at 0.76 when the ear leaves underwent SG‐MSC preprocessing at the R1 stage. Similarly, the correlation coefficients for the CIgreen and GNDVI indices were maximized at 0.76 when lower leaves with SG‐SNV‐DT preprocessing. SNV, SNV‐DT, and MSC preprocessing based on the SG methods, the seven vegetation indices (CVI, CVI2, DVI, NDVI, PVI, RVI, and VIG) exhibited significantly enhanced correlations with SPAD values.

In summary, applying SG smoothing and the preprocessing techniques SNV, SNV‐DT, and MSC significantly improved the correlation between vegetation indices and SPAD values, thereby enhancing the accuracy of models that estimate leaf SPAD values. Vegetation indices derived from the fully expanded apical leaves at the V12 stage and the ear leaves at the R1 stage generally exhibited stronger correlations with SPAD values than indices derived from other leaves.

### 3.4 Construction of SPAD Prediction Model for Maize Leaves

#### 3.4.1 Construction of Leaf SPAD Inversion Model Based on Single Vegetation Index

Based on the correlation coefficients between the vegetation indices and leaf SPAD of the fully expanded apical leaf at V12 stage, and the three ear leaves at R1 stage, the vegetation indices and preprocessing methods yielding the highest correlation coefficients (Table 5) were selected to establish the predictive model for maize leaf SPAD values, employing linear functions, quadratic polynomial functions, exponential functions, and power functions respectively.

**TABLE 5:** Optimal vegetation indices and preprocessing methods.

Stages	Leaf position	Optimal vegetation indices	Optimal preprocessing methods
V12	The fully expanded apical leaf	RVI (−0.78)	SG‐SNV
R1	The upper ear leaf	NDRE (0.74)	SG‐MSC
	The ear leaf	NDRE (0.76)	SG‐SNV
	The lower ear leaf	SAVI (−0.75)	SG‐SNV‐DT

**FIGURE 6:**
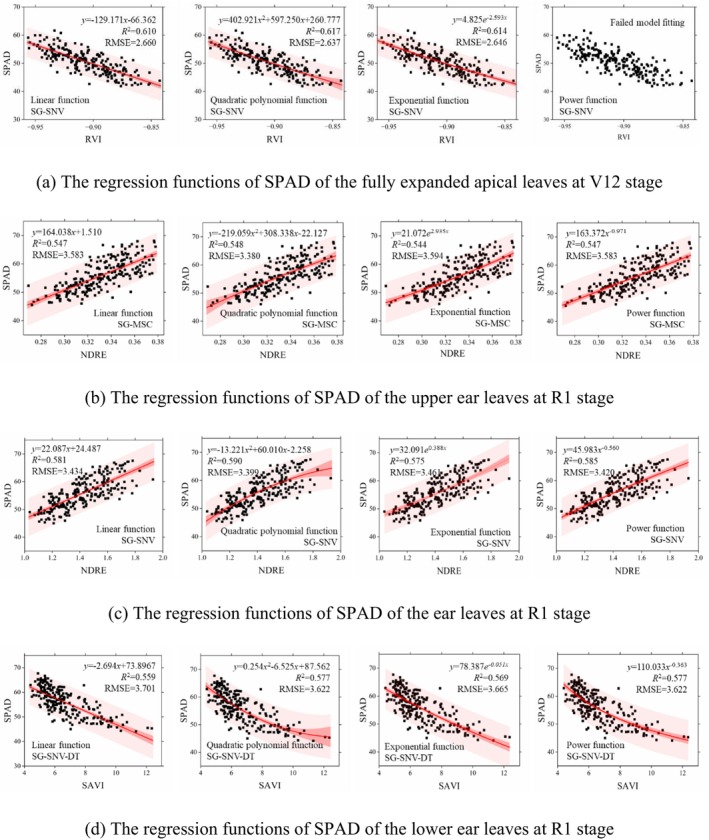
Regression functions of SPAD of (a) the fully expanded apical leaves at V12 stage, (b) the upper ear leaves, (c) the ear leaves, and (d) the lower ear leaves at R1 stage.

The SPAD value regression functions based on single vegetation index under different preprocessing methods are shown in Figure 6. The *R* ^2^ for the first three functions all exceeded 0.6 at V12 stage. The quadratic polynomial model yielded the highest *R* ^2^ of 0.617 and the lowest RMSE of 2.637, indicating optimal model fit and minimal prediction error for SPAD values. When employing power functions for modeling, the data could not be fitted, suggesting that a stable solution could not be achieved under this functional form.

SPAD prediction results for the upper ear leaves at R1 stage revealed that all four functional models based on SG‐MSC‐NDRE achieved *R* ^2^ exceeding 0.5. Among these, the quadratic polynomial model demonstrated the highest *R* ^2^ of 0.548 and the lowest RMSE of 3.580. The linear and power models exhibited comparable performance, slightly outperforming the exponential model. During the R1 stage, the quadratic polynomial model based on the NDRE index achieved the highest *R* ^2^ (0.590) and the lowest RMSE (3.399) for SPAD prediction of ear leaves under SG‐SNV preprocessing. The linear and power function models demonstrated comparable performance, with both slightly outperforming the exponential function model. Modeling based on SAVI under SG‐SNV‐DT pretreatment yielded the highest *R* ^2^ (0.587) and the lowest RMSE (3.412) for SPAD prediction of lower panicle leaves using the quadratic polynomial and power function models, which demonstrated optimal data fitting and minimal prediction error for SPAD values.

#### 3.4.2 Construction of Leaf SPAD Inversion Model Based on Multi Vegetation Indices

The Sample Pairwise‐Nearest‐Neighbor Partitioning (SPXY) algorithm was employed to partition the dataset into a 4:1 training‐to‐test ratio, with 21 vegetation indices and SPAD that had been preprocessed by different methods used as model inputs and outputs. RF model was constructed in order to predict maize leaf SPAD values, with the results of this analysis presented in Table 6. The model demonstrated an *R* ^2^ value greater than 0.60 across the test set, with an RPD that remained above 1.8 and a maximum value of 1.97, thus enabling relatively precise prediction of maize leaf SPAD values.

**TABLE 6:** Prediction results of RF model of leaf SPAD value.

Stages	Leaf position	Preprocessing method	Training set			Test set		
			*R* ^2^	RMSE	RPD	*R* ^2^	RMSE	RPD
V12	The fully expanded apical leaf	MSC	0.83	1.77	2.41	0.71	2.35	1.87
		SNV	0.83	1.74	2.46	0.68	2.39	1.78
		SNV‐DT	0.81	1.83	2.29	0.68	2.61	1.77
R1	The lower ear leaf	MSC	0.82	2.27	2.35	0.64	3.19	1.69
		SNV	0.80	2.41	2.24	0.68	2.69	1.80
		SNV‐DT	0.81	2.31	2.31	0.67	3.07	1.75
	The ear leaf	MSC	0.82	2.20	2.36	0.69	3.22	1.82
		SNV	0.84	2.13	2.48	0.63	3.29	1.67
		SNV‐DT	0.83	2.18	2.44	0.63	3.18	1.66
	The upper ear leaf	MSC	0.82	2.31	2.40	0.68	3.23	1.80
		SNV	0.81	2.30	2.28	0.64	3.41	1.97
		SNV‐DT	0.81	2.35	2.32	0.69	3.40	1.82

The RPD of vegetation indices modeled using three distinct preprocessing methods exceeded 1.4 in the test set at the V12 stage. Among these, the vegetation index preprocessed via MSC yielded the RPD of test set modeling exceeding 1.8, thereby demonstrating superior predictive capability with an *R* ^2^ of 0.71 and the lowest RMSE of 2.35. At the R1 stage, models constructed using vegetation indices from upper and lower ears subjected to SNV preprocessing demonstrated optimal performance. *R* ^2^, RMSE and RPD of the test set were found to be 0.68, 2.69, 1.80, and 0.74, 3.41, and 1.97 respectively. For the ear leaves, vegetation indices modeled using MSC‐preprocessed data yielded optimal results, with training and test set *R* ^2^, RMSE, and RPD values of 0.82, 2.20, and 2.36, and 0.69, 3.22, and 1.82, respectively.

The analysis of RF modeling results suggested that the RF models based on vegetation indices demonstrated variable performance in response to divergent preprocessing methodologies. Models based on vegetation indices derived from the fully expanded apical leaves at the V12 stage and ear leaves at the R1 stage under MSC preprocessing demonstrated the most favorable performance. Conversely, models utilizing vegetation indices from the upper and lower ear leaves under SNV preprocessing yield optimal results.

## 4 Discussion

### 4.1 Analysis of Sensitive Leaf Positions and Stages in Maize

The vertical distribution of nitrogen nutrition represented an inherent and very significant change rule of crops, which should be fully considered when carrying out spectral diagnosis of chlorophyll level and exploring the quantitative relationship between them (Li and Wang 2013). When natural light illuminated the upper leaves of crop canopy, most of the light energy was either reflected into the atmosphere or transmitted through the upper foliage into the middle and lower layers (Yin and Struik 2015). Consequently, the distribution of chlorophyll levels, which characterized crop photosynthetic efficiency, was inherently uneven within the canopy, exhibiting distinct vertical distribution patterns (Winterhalter et al. 2012). As a theory concerning the vertical distribution of nutrients between crop leaf layers both domestically and internationally, optimization theory proposed that crops nutrients were prioritize allocated to areas with higher light energy acceptance rates, meaning nitrogen efficiency in upper leaves was significantly higher than in middle and lower leaves (Hirose and Werger 1987). At present, the quantitative diagnosis of chlorophyll level spectrum with canopy leaves as the analysis object had achieved good results (Zhao et al. 2023). However, the distribution of light in the crop canopy had been confirmed to be the inverted triangle structure, which indicated that the upper and middle crop leaves were more conducive to receiving sufficient photosynthetically active radiation (Bertheloot et al. 2008).

Maize was a representative crop with a high nitrogen demand, characterized by tall stature, large number of leaves, and the substantial area of each individual leaf (Li, Ming, et al. 2022; Song et al. 2024). The chlorophyll content exhibited significant spatial and temporal variability between different leaf positions of the same plant. The specific performance was as follows: the vertical variation characteristics of SPAD values in the various leaf layers of maize were consistent with the trend of photosynthetic pigments and nitrogen nutrition indexes in corresponding leaf positions, with “bell‐shaped” variation characteristic. Therefore, the highest photosynthetically active radiation and material synthesis efficiency were neither in the top layer nor the middle and lower layers, but rather in the middle and upper leaves. The fully expanded apical leaves of the vegetative growth stage and the three ear leaves of the reproductive growth stage belonged to the middle and upper leaves and played a key role in the growth and yield formation of maize in the corresponding stage.

Accordingly, the dynamic growth effects of leaf SPAD value were systematically expounded in this study, which showed the SPAD of maize leaves at different stages exhibited significant variation. The SPAD value of the fully expanded apical leaves of the vegetative growth stage decreased as the growth stage advanced. The SPAD value of ear leaves in the reproductive growth stage exhibited a gradual increase with the progression of the growth stage, manifesting an initial decrease followed by an increase. During the maize growth stages from V6 to V12 stages, the plants grew rapidly, and more nutrients were supplied to the plants for longitudinal growth. The distribution of plant resources changed, resulting in a decrease in SPAD value at the V12 stage. In the R1 and R2 stage, the leaf area of ear leaves was larger than that of others, and the photosynthetic efficiency was higher, which bore more nutrient transport tasks to meet the needs of grain filling, so the SPAD value of leaves increased.

### 4.2 Analysis of SPAD Spectral Inversion Methods in Maize Leaf

The combination of pretreatment methods and vegetation indices were commonly used in the spectral inversion analysis of crop chlorophyll content, enhancing the precision of crop chlorophyll levels prediction model. A variety of spectral preprocessing methods, including SG, MSC, and SNV, were employed to reduce or eliminate redundant information in hyperspectral data. The vegetation indices were utilized to obtain effective spectral features. Consequently, four preprocessing methods and 21 spectral vegetation indices were used to analyze the spectral reflectance of typical leaf positions in sensitive stages. The correlation analysis results of vegetation indices and leaf SPAD indicated that the correlation coefficient of vegetation indices after pretreatment had been significantly improved. Taking AVSI of the fully expanded apical leaves at V12 stage as an illustration, the correlation coefficients of SG‐SNV, SG‐SNV‐DT, and SG‐MSC were −0.67, −0.72, and −0.67, respectively, which were significantly higher than those (−0.02) of the SG treatment.

A well‐designed model could effectively improve the accuracy of chlorophyll content estimation. Maize chlorophyll level estimation models contained two approaches: The first was the functional regression model based on single vegetation index, the second was the machine learning model utilizing multispectral vegetation indices (Li, Li, et al. 2023; Croft et al. 2020). Functional equations enabled rapid establishment of chlorophyll level estimation models, but simple functions struggled to accurately derive leaf information. Machine learning possessed unique advantages in growth parameter inversion due to its exceptional adaptive self‐learning capabilities and nonlinear mapping abilities, aiding in the revelation of multiple intrinsic relationships between spectral indices and SPAD values (Berger et al. 2021). RFR models exhibited high accuracy and strong robustness against noise, widely applied in solving nonlinear problems, which could identify complex nonlinear relationships between leaf SPAD and spectral indices (Müllerova et al. 2021). This study employed RFR approach to construct prediction models based on multiple vegetation indices. The results demonstrated the ability to meet the demand for rapid and precise monitoring of leaf SPAD. However, variations in model accuracy were observed, which primarily stemmed from differences in leaf sensitivity to SPAD across different growth stages.

### 4.3 Limitations and Prospects of This Study

1. SPAD value was selected as a representative measurement of leaf chlorophyll content, which was significantly positively correlated with leaf nitrogen content, and could quickly characterize the nutritional status and photosynthetic physiological level of maize leaves. Appropriate chlorophyll and nitrogen accumulation could improve photosynthetic efficiency, ensure dry matter accumulation, and thus support yield formation. However, SPAD value could not maintain a strict and consistent change with maize nutrient concentration and actual yield, due to the effects of nitrogen redundancy, growth stage replacement, leaf senescence process and field environmental heterogeneity. Therefore, the mathematical relationship between SPAD value and maize nitrogen content and yield in complex field environment needs further study.
2. The tested maize varieties used in this study were Zhengdan 958 and Yuke 918. Zhengdan 958 was “N‐sensitive and high‐accumulation” variety, which nitrogen utilization rate changed significantly with the amount of nitrogen applied, showing strong nitrogen absorption and transport capacity. Yuke 918 belonged to the type of “high nitrogen tolerance and gentle response to nitrogen.” The nitrogen uptake is slightly lower, and the nitrogen utilization rate changes slowly with the nitrogen application rate. The two varieties had significant differences in nitrogen response. It should be noted that due to the absolute dominance of hybrids in the current high‐yield maize production system, there are significant genotypic differences in the genetic background, nitrogen efficiency and plant nitrogen response characteristics of different hybrids. Therefore, the results of this study may be difficult to fully reveal the overall response mode of maize to changes in nitrogen availability in field production, and it is still necessary to expand the variety type for further verification.

## 5 Conclusions

The fully expanded apical leaves of the vegetative growth stage and the three ear leaves of the reproductive growth stage were studied to explore the dynamic changes of SPAD at typical leaf positions in different periods of maize and analyze the sensitive stage and leaf position suitable for SPAD monitoring of maize leaves.

1. The SPAD of maize leaves at different growth stages was significantly different. The SPAD of the fully expanded apical leaves of the vegetative growth stages decreased with the advance of the growth stage. The SPAD of the ear leaves in the reproductive growth stage gradually increased with the advance of the growth stage, and the overall trend was first decreased and then increased.
2. The twelve‐leaf (V12) stage and the silking (R1) stage were determined to be the sensitive stages, in accordance with the correlation analysis of SPAD and spectral reflectance of maize leaves. The strongest correlation (−0.78) at the V12 stage was observed between SG‐SNV and RVI combination, and the strongest correlation (0.76) for the ear leaves at the R1 stage was found between SG‐SNV and NDRE combination.
3. For the single vegetation index modeling, the quadratic polynomial function yielded the best performance, in which the regression function *R* ^2^ = 0.617, RMSE = 2.637 at the V12 stage, and the regression function *R* ^2^ = 0.590, RMSE = 3.399 at the R1 stage. For the RF model, the best prediction data (*R* ^2^ = 0.71, RMSE = 2.35, RPD = 1.87) was showed at the V12 stage after MSC pretreatment. The prediction effect of the ear leaves after MSC pretreatment was slightly better (*R* ^2^ = 0.69, RMSE = 3.22, RPD = 1.82) than the upper and lower ear leaves. Therefore, it is recommended to monitor the SPAD value of maize leaves at the V12 and R1 stages, and then make nitrogen fertilizer management decisions to increase maize yield.

## Author Contributions

**Fu Zhang:** writing – review and editing, project administration. **Baoping Yan:** writing – original draft, visualization. **Le Yang:** investigation, formal analysis. **Fangyuan Zhang:** software, data curation. **Yakun Zhang:** validation. **Yafei Wang:** project administration. **Shaukat Ali:** writing – review and editing, validation. **Sanling Fu:** conceptualization, supervision.

## Funding

This work was supported by the Henan Province Key Research and Development project, 251111110800, National Natural Science Foundation of China, 32501779, 32572197, the Scientific and Technological Project of Henan Province, 252102230034, 242102110337, Graduate Education Reform Project of Henan Province, 2023SJGLX180Y, Basic Research Program of Jiangsu, BK20250866, Jurong City Science and Technology Plan Project, ZA32410, and Leading Talents in Science and Technology Innovation of Luoyang City.

## Ethics Statement

The authors have nothing to report.

## Conflicts of Interest

The authors declare no conflicts of interest.

## Data Availability

The data that support the findings of this study are available from the corresponding author upon reasonable request.
